# The role of a hepatitis C virus vaccine: modelling the benefits alongside direct-acting antiviral treatments

**DOI:** 10.1186/s12916-015-0440-2

**Published:** 2015-08-20

**Authors:** Nick Scott, Emma McBryde, Peter Vickerman, Natasha K. Martin, Jack Stone, Heidi Drummer, Margaret Hellard

**Affiliations:** Centre for Population Health, Burnet Institute, 85 Commercial Rd, Melbourne, VIC 3004 Australia; Department of Epidemiology and Preventive Medicine, Monash University, Clayton, VIC 3800 Australia; Doherty Institute for Infection and Immunity, 792 Elizabeth Street, Melbourne, VIC 3000 Australia; Department of Medicine, The University of Melbourne, Parkville, VIC 3050 Australia; School of Social and Community Medicine, University of Bristol, Bristol, UK; Department for Global Health and Development, London School of Hygiene and Tropical Medicine, London, UK; Division of Global Public Health, University of California, San Diego, USA; Centre for Biomedical Research, Burnet Institute, 85 Commercial Rd, Melbourne, VIC 3004 Australia; Department of Microbiology, Monash University, Clayton, VIC 3800 Australia; Department of Microbiology and Immunology, The University of Melbourne at the Peter Doherty Institute for Infection and Immunity, Melbourne, VIC 3000 Australia; Australian Institute of Tropical Health and Medicine, James Cook University, Townsville, QLD 4811 Australia

## Abstract

**Background:**

Hepatitis C virus (HCV) elimination is being seriously considered globally. Current elimination models require a combination of highly effective HCV treatment and harm reduction, but high treatment costs make such strategies prohibitively expensive. Vaccines should play a key role in elimination but their best use alongside treatments is unclear. For three vaccines with different efficacies we used a mathematical model to estimate the additional reduction in HCV prevalence when vaccinating after treatment; and to identify in which settings vaccines could most effectively reduce the number of treatments required to achieve fixed reductions in HCV prevalence among people who inject drugs (PWID).

**Methods:**

A deterministic model of HCV transmission among PWID was calibrated for settings with 25, 50 and 75 % chronic HCV prevalence among PWID, stratified by high-risk or low-risk PWID. For vaccines with 30, 60 or 90 % efficacies, different rates of treatment and vaccination were introduced. We compared prevalence reductions achieved by vaccinating after treatment to prevent reinfection and vaccinating independently of treatment history in the community; and by allocating treatments and vaccinations to specific risk groups and proportionally across risk groups.

**Results:**

Vaccinating after treatment was minimally different to vaccinating independently of treatment history, and allocating treatments and vaccinations to specific risk groups was minimally different to allocating them proportionally across risk groups. Vaccines with 30 or 60 % efficacy provided greater additional prevalence reduction per vaccination in a setting with 75 % chronic HCV prevalence among PWID than a 90 % efficacious vaccine in settings with 25 or 50 % chronic HCV prevalence among PWID.

**Conclusions:**

Vaccinating after treatment is an effective and practical method of administration. In settings with high chronic HCV prevalence among PWID, even modest coverage with a low-efficacy vaccine could provide significant additional prevalence reduction beyond treatment alone, and would likely reduce the cost of achieving prevalence reduction targets.

**Electronic supplementary material:**

The online version of this article (doi:10.1186/s12916-015-0440-2) contains supplementary material, which is available to authorized users.

## Background

Hepatitis C virus (HCV) elimination is now being seriously considered globally [1–3]. In developed countries, people who inject drugs (PWID) are at greatest risk of HCV infection [4–6]. The prevalence of HCV RNA among PWID varies globally, ranging from 10 to 97 % [6, 7] and is estimated to be greater than 50 % in many countries. For this reason elimination models have focused on this group, using a combination of treatment as prevention and harm reduction, including opioid substitution therapy (OST) and needle and syringe programmes (NSPs) [8, 9]. The advent of highly effective direct-acting antiviral (DAA) treatment, with 90 % cure rates, improved tolerability and a comparably short duration of therapy (up to 12 weeks) [10–12] has increased optimism about achieving elimination. A number of fixed-dose combinations such as sofosbuvir and ledipasvir [10–13], and paritaprevir/ritonavir/ombitasvir and dasabuvir [14], have been licenced by the US Food and Drug Administration and other countries’ regulatory bodies, with a number of other drug combinations in phase 3 clinical trials. At the same time, there is real concern that the high cost of DAAs and large numbers of people requiring treatment make current elimination strategies prohibitively expensive, reducing the likelihood of substantial progress in HCV prevalence reduction in the next 15 years.

Vaccines typically represent the most cost-effective strategy to prevent infectious disease and development is continuing [15]. HCV is a challenge for vaccine development owing to its high sequence variation, resulting in the need for a vaccine to provide broad protection against the seven genotypes and >50 subtypes circulating worldwide. Several prophylactic vaccines are in clinical and preclinical development, the most advanced of which is the T cell-based approach. This employs a chimpanzee adenovirus prime followed by a modified vaccinia Ankara virus boost strategy encoding the NS3, NS4, NS5A and NS5B proteins of genotype 1b, which generate long-lived, broad T cell responses. It is now in phase 1/2 clinical trials to determine efficacy in preventing chronic HCV [15]. Alternative vaccine strategies use the viral glycoproteins to generate neutralizing antibody responses. These include a gpE1/E2 vaccine [16, 17] tested in a phase 1 clinical trial in humans and demonstrating some cross-neutralization capacity, and others in preclinical development [18–21]. However, the availability of highly effective HCV treatments has raised questions as to what benefits a HCV vaccine may provide, particularly if it were not 100 % effective as current research suggests [22, 23].

HCV vaccines are likely to play an important role in HCV elimination but it is unclear how best to use them alongside highly effective, but costly, HCV treatments. With this in mind, we explored the possible benefit of three different HCV vaccines in different epidemic settings when introduced at the same time as scaled-up treatment. Administering a vaccination after treatment could be a useful strategy for limiting reinfection and is practical because the HCV RNA status of the patients is already known. In addition, it could be an effective way of targeting higher-risk individuals because they are more likely to be infected.

In this study we consider settings with low (25 %), medium (50 %) or high (75 %) HCV RNA prevalence among PWID. Distinguishing the presence of HCV RNA (current HCV infection) from HCV antibodies in individuals, which could be present owing to either acute, chronic or resolved infection, requires a polymerase chain reaction test that is rarely performed in epidemiological studies. Countries with low HCV antibody prevalence among PWID include Czech Republic (25 %), Tanzania (22 %) and Uruguay (22 %); countries with medium HCV antibody prevalence among PWID include Argentina (54 %), Australia (55 %), Iran (50 %), the UK (51 %) and Uzbekistan (52 %); and countries with high HCV antibody prevalence among PWID include France (74 %), Indonesia (77 %), the USA (73 %) and Vietnam (74 %) [6]. However, the prevalence of HCV antibodies may be up to 25 % higher than the prevalence of HCV RNA [24]. In each of the three HCV RNA prevalence settings, we considered three different vaccines, which had low (30 %), medium (60 %) and high (90 %) efficacies. In each scenario, a deterministic model of HCV transmission among PWID was used to predict (1) the additional prevalence reduction that could be achieved by vaccinating after treatment and whether this would be as effective as a community-based vaccination programme; (2) how much additional efficiency could be gained by targeting both vaccinations and treatments at either high or low injecting risk PWID; and (3) under what circumstances a scaled-up vaccine programme could most effectively reduce the number of treatments required to achieve fixed prevalence reduction targets.

## Methods

### Model description

**Fig. 1:**
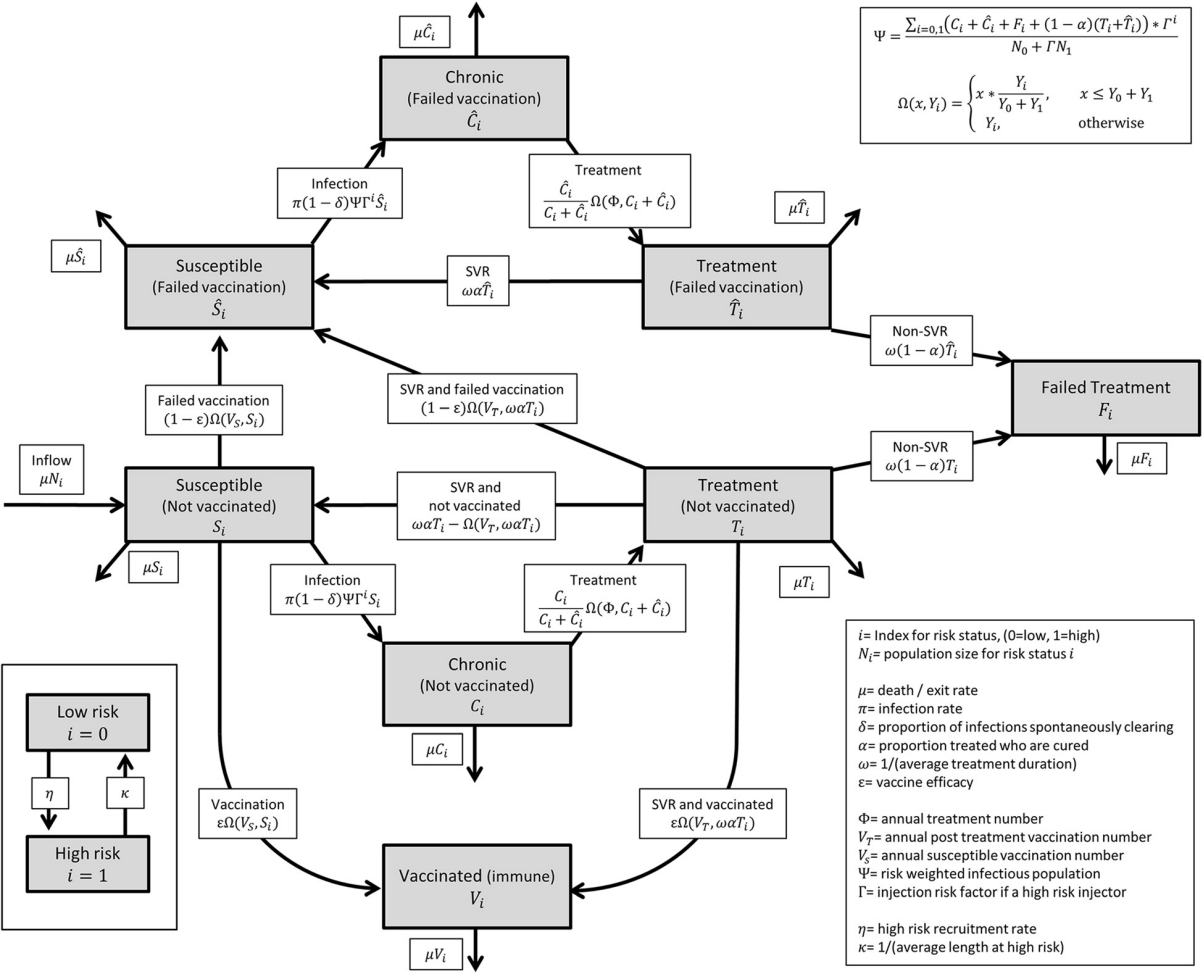
Model schematic

We used a modified version of the open deterministic compartment model of HCV transmission among PWID from Martin et al. [25] shown in Fig. 1, assuming a prophylactic vaccine with efficacy ε was available that offered a duration of immunity greater than the length of injecting career and perfect protection for a proportion ε of PWID vaccinated, and no protection for the remaining (1−ε) who ‘fail vaccination’.

PWID were distinguished as either susceptible and not vaccinated (*S*, HCV RNA- population, including infection-naïve PWID or PWID achieving a sustained viral response [SVR] either spontaneously or through treatment); chronically infected and not vaccinated (*C*, PWID who do not spontaneously clear their infection, including both initial infections and reinfections); in treatment and not vaccinated (*T*); vaccinated and immune (*V*); susceptible after failing vaccination (*Ŝ*); chronically infected after failing vaccination (*Ĉ*); in treatment after failing vaccination ($\widehat{T}$); or chronically infected after failing treatment (*F*). Each compartment was stratified by injecting risk (high versus low, *i* = 1 or 0 respectively, with risk definition based on the proportion of PWID experiencing unstable housing—see Table 1; populations mixed proportionally), with the infectivity of high-risk PWID increased by a factor Γ. PWID at high risk remained there for an average duration (1/κ), and the total size of the high-risk population was held fixed and calibrated by varying the recruitment parameter η. Estimates for Γ, κ and the size of the high-risk population can be found in Table 1.

**Table 1:** Model parameters and references

Parameter	Symbol	Value	References
Duration of injecting career	1/μ_1_	14 years	Fazito et al. global average [26]
Mortality rate	μ_2_	0.0083 per year	Stoové et al. [27]
Exit rate	μ	μ_1_ + μ_2_ per year	
Proportion at high risk, defined as the proportion of PWID experiencing unstable housing [16]	Vary η to calibrate	0.17	O’Keefe et al. [28]
Duration at high risk	12/κ	13 months	O’Keefe et al. [28]
Recruitment to high risk	η	Calibrated to proportion at high risk	
Infection risk factor of high-risk PWID compared to low-risk PWID	Γ	3.6	Turner et al. [29], Allen et al. [30], Aitken et al. [31]
Infection rate	π	Calibrated to initial prevalence	
Proportion of infected who spontaneously clear	δ	0.26	Micallef et al. [24]
Proportion treated who are cured (interferon-free DAAs, all genotypes)	α	0.9	Lawitz et al. [10], Gane et al. [11], Poordad et al. [12]
Treatment duration (interferon-free DAAs, all genotypes)	52/ω	12 weeks	Lawitz et al. [10], Gane et al. [11], Poordad et al. [12], Chen et al. [32]
Vaccine efficacy	ε	30 %, 60 %, 90 %	Assumed
Vaccine duration of protection		Greater than the length of injecting career	Assumed

*DAA* direct-acting antiviral, *PWID* people who inject drugs

PWID leave each compartment owing to cessation of injecting or death at a fixed rate μ and the total population is held constant by entry of new PWID who are assumed to be unvaccinated and susceptible, with a fixed proportion being high risk. Both vaccine-naïve susceptible PWID and those who failed vaccination become infected at a rate proportional to an infection rate π, the risk-weighted number of susceptible PWID (with risk weighting Γ for PWID at high risk), and the risk-weighted proportion Ψ of PWID either currently infected or in treatment and not achieving SVR. A proportion (1−δ) of newly infected PWID fail to spontaneously clear the virus and become chronically infected. A fixed annual number *Φ* of treatment-naïve or reinfected PWID (*C* and *Ĉ*) are recruited into treatment (or all of them if *C* + *Ĉ* < *Φ*) selected proportionally from unvaccinated and failed vaccination chronically infected compartments (*Ci* and *Ĉi*) and across risk levels (*i* = 0, 1). Treatment is completed after an average duration 1/ω years, and the proportion (1−α) not achieving SVR move to the failed treatment compartment where they remain and are not retreated. Given the current limited data on retreatment outcomes following treatment failure with DAAs, the low numbers this is likely to represent, and current limits on treatment numbers, we believe this is the most practical way to manage this group in the model. The proportion α achieving SVR are either vaccinated (of which ε becomes immune and [1−ε] fail vaccination and move to *Ŝ*) or returned to their respective susceptible pools. A fixed annual number V_T_ of vaccine-naïve susceptible PWID (or all of S if S < V_T_) are vaccinated each year. Both injecting risk states are equally eligible for vaccination.

The model was run to equilibrium without any treatments or vaccinations and the initial conditions (if non-zero) do not affect the steady-state values; equilibrium prevalence and the proportion at high risk were calibrated by varying the infection rate π and high-risk recruitment rate η respectively to achieve the desired scenario-dependent baseline conditions. After steady-state with no treatments or vaccinations was reached, (maximum) treatment and vaccination numbers were introduced simultaneously. Model parameters were taken from previously published studies [10–12, 24, 26–32], with treatment parameters updated to reflect the latest trial data on DAAs. These are provided with references in Table 1.

Natural immunity following spontaneous clearance of the virus was not included in the model, because although this has been observed in humans [33, 34] and chimpanzees [35], the percentage of patients likely to experience immunity, and for how long, is currently unclear. Previous modelling studies testing this feature found minimal effects on model outcomes [36]; in particular, omission of this feature will lead to slightly lower estimates for the infection parameter π used to calibrate prevalence, and conservative estimates for the benefits of vaccines in preventing transmission. Similarly, immunity following SVR was not included in the model. Although the extent to which it occurs following treatment with DAAs is currently unclear, we have assumed it is similar to that following previous HCV treatments. This is again a conservative assumption and means that estimates of prevalence reduction due to treatment may be understated.

### Vaccination after treatment versus community-based vaccination

To determine the additional prevalence reduction a vaccine could provide if administered as part of a treatment regimen, for all combinations of vaccine efficacies of 30, 60 and 90 %; initial chronic HCV prevalence among PWID of 25, 50 and 75 %; and treatment numbers of 10, 20 and 40/1,000 PWID per year (treated independent of risk), the 15-year relative prevalence reduction was calculated and compared for (1) no vaccination; (2) vaccinating all who were treated and had a SVR; (3) vaccinating susceptible PWID (in equal numbers to successful treatments, i.e. 90 % of those treated, independent of risk); and (4) vaccinating all susceptible PWID and all who were treated and had a SVR. The fourth category would be unrealistic in practice but provides an upper bound for the maximum relative prevalence reduction that could be achieved.

### Maximizing efficiency by treating and vaccinating specific risk groups

For all combinations of vaccine efficacies of 30, 60 and 90 %; initial chronic HCV prevalence among PWID of 25, 50 and 75 %; treatment numbers of 10, 20 and 40/1,000 PWID per year; and vaccination numbers of 10, 20 and 40/1,000 susceptible PWID per year, each scenario was run multiple times as the proportion of treatments and the proportion of vaccination courses administered to high-risk PWID took the values 0, 0.2, 0.4, 0.6, 0.8 and 1. Relative prevalence reduction was measured after 15 years for each run and the ‘most efficient’ and ‘least efficient’ methods for allocating treatments and vaccinations were defined as the ones achieving the maximum and minimum, respectively, relative prevalence reduction.

Relative prevalence reduction after 15 years using the most efficient method was compared to selecting proportionally across risk groups both chronically infected PWID for treatment and susceptible PWID for vaccination for all combinations of vaccine efficacies, initial prevalence, treatment numbers and vaccination numbers. This represents the maximum theoretical benefit in prevalence reduction obtained by allocating treatments and vaccinations most efficiently to injecting risk groups.

### Reaching fixed prevalence reduction targets: simultaneously introducing a vaccination programme to reduce treatment numbers

Treatment rates of between 0 and 80/1,000 PWID per year and simultaneous vaccinations rates of between 0 and 200/1,000 susceptible PWID per year were simulated, with both treatments and vaccinations allocated proportionally across risk groups. Each simulation was rerun for vaccine efficacies of 30, 60 and 90 % and initial chronic prevalence of 25, 50 and 75 %, and relative prevalence reduction was measured every five years for a 30-year period. Linear regression was used to estimate the relationship between vaccination numbers and treatment numbers required to achieve 25, 50 and 75 % relative prevalence reductions after 10, 15 and 30 years for each scenario. These estimates can be interpreted as measuring the effectiveness of a vaccine at reducing the number of treatments required to maintain prevalence reduction targets.

### The basic reproduction number

The basic reproduction number (R0)—the average number of new infections caused by one typical infected individual in a completely susceptible population—is an extremely important value for infectious diseases. Values of R0 < 1 indicate that the disease-free state is asymptotically stable, and so the disease is expected to eventually become extinct, while values of R0 > 1 indicate that the disease-free state is asymptotically unstable and the disease is able to invade a population [37]. Hence, the existence of equilibrium prevalence in our model at baseline (with no imported infections) indicates that R0 > 1. Interventions that are able to reduce R0 from above one to below one will have the most significant impacts—for our model this can be done with a vaccine to reduce the number of susceptible individuals in the population (i.e. increasing the ‘herd immunity’ to a point where HCV can no longer persist).

For settings with 25, 50 and 75 % initial chronic HCV prevalence among PWID (and hence different values for the calibrated transmission parameter π), R0 was calculated [38, 39], and in each case the proportion of susceptible PWID that would need to be vaccinated to reduce R0 from above one to below one was determined. Details of these calculations are provided in Additional file 1: Supplementary material.

### Sensitivity of parameters

In separate scenarios, the infectivity of the high-risk population was doubled from 3.6 to 7.2, the proportion of PWID at high risk was doubled from 0.17 to 0.34, the duration of injecting career was halved from 14 years to 7 years, the average length of time at high risk was increased to 14 years (the length of injecting career), and fully assortative mixing amongst injecting risk groups (e.g. high-risk PWID only infect/are infected by high-risk PWID) was assumed. A scenario where the vaccine efficacy was halved if administered after treatment was also explored as follows. For scenarios where only susceptible PWID were vaccinated (i.e. not as part of treatment), the overall vaccine efficacy was modified according to movements into the susceptible compartment, namely:

$$ \varepsilon (t)=\widehat{\varepsilon}*\frac{\mu \left({N}_0+{N}_1\right)+\frac{1}{2}\omega \alpha \left({T}_0+{T}_1\right)}{\mu \left({N}_0+{N}_1\right)+\omega \alpha \left({T}_0+{T}_1\right)}, $$

where $\widehat{\varepsilon}$ is the stated efficacy. Here, we have implicitly used time-dependent movements to approximate the composition of the susceptible compartment as either previously treated or treatment-naïve. Under each of the alternate parameter assumptions, the analyses comparing vaccination after treatment to vaccination in the community and maximizing efficiency by treating and vaccinating specific risk groups were repeated.

## Results

### Vaccination after treatment versus community-based vaccination

**Fig. 2:**
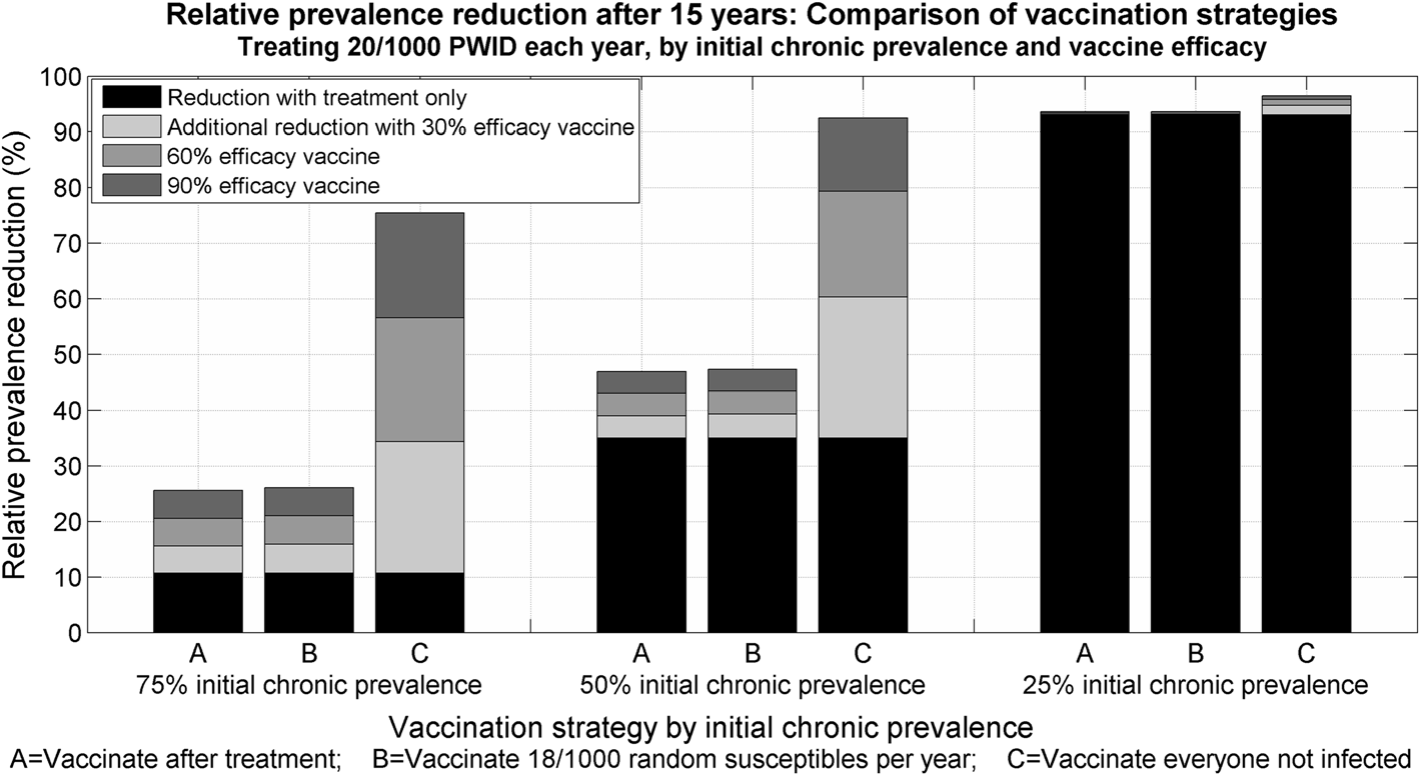
Vaccination strategy: comparison of relative prevalence reduction using fixed treatment numbers of 20/1,000 PWID per year and vaccinating all who were treated and had a SVR (*A*); vaccinating an equivalent number independently of treatment history in the community (*B*); and vaccinating everyone not infected (*C*). Settings with 75, 50 and 25 % initial chronic HCV prevalence among PWID, vaccines with 30, 60 and 90 % efficacies. *HCV* hepatitis C virus, *PWID* people who inject drugs

The 15-year relative prevalence reduction when 20/1,000 PWID were treated each year was compared in settings with 25, 50 and 75 % initial chronic HCV prevalence when (1) everyone was vaccinated after successful treatment; (2) 18/1,000 PWID were vaccinated in the community each year; and (3) everyone not chronically infected and not previously vaccinated was vaccinated each year with different efficacy vaccines (30, 60 and 90 %) (Fig. 2). Vaccinating after treatment was as effective at reducing prevalence as vaccinating an equivalent number of PWID in the community. This remained true for other treatment numbers considered (see Additional file 1: Figure S1). A vaccine provided greater benefit in a setting with high chronic HCV prevalence among PWID; in a setting with low chronic HCV prevalence among PWID, modest treatment numbers were sufficient to virtually eliminate the epidemic.

### Improving efficiency by treating and vaccinating specific risk groups

For settings with low or medium chronic HCV prevalence among PWID, the most efficient method was to treat and vaccinate high-risk PWID. For a setting with high chronic HCV prevalence among PWID, the most efficient method was to treat low-risk PWID and vaccinate high-risk PWID. The difference in a setting with high chronic HCV prevalence among PWID can be attributed to pre-emptive saturation [40] of HCV infection among high-risk PWID; enough contacts are ‘wasted’ on already infected PWID that it becomes more beneficial to treat low-risk PWID.

**Fig. 3:**
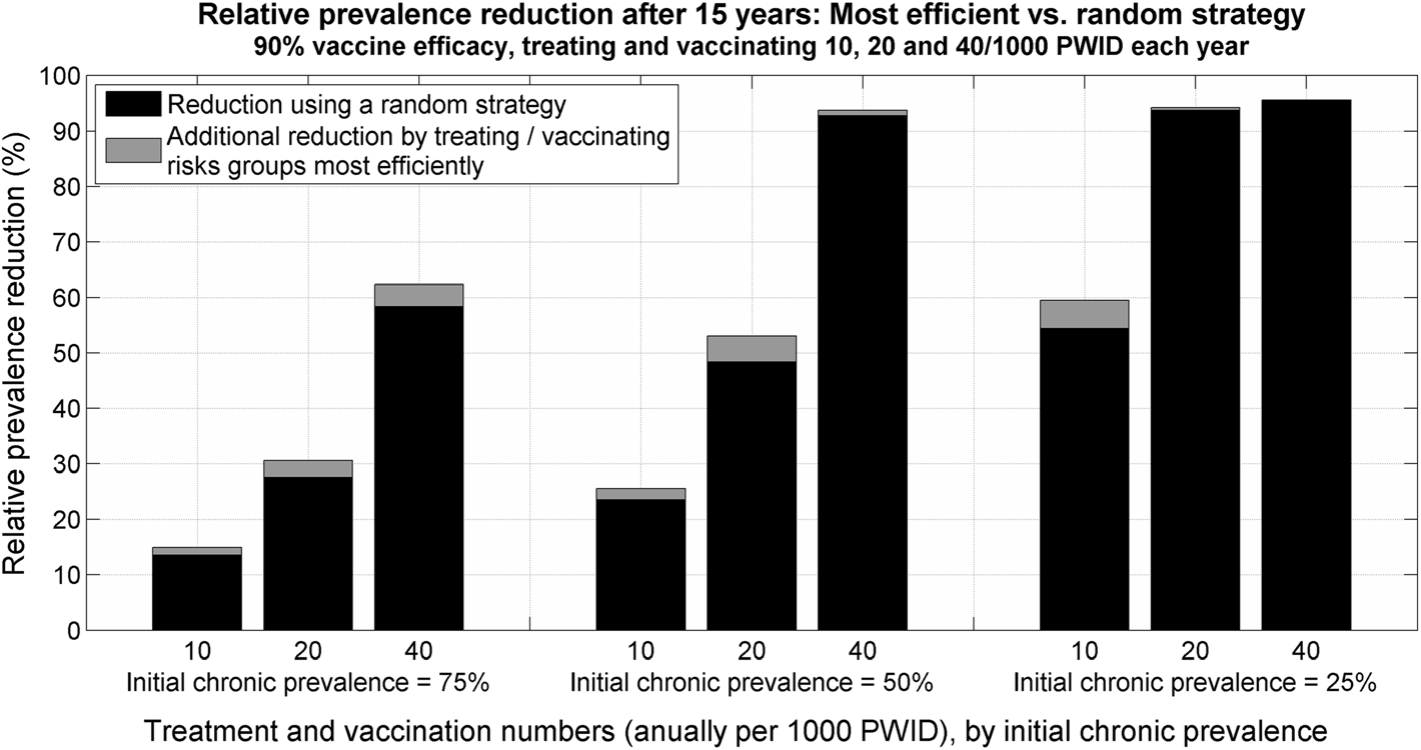
Risk allocation: comparison of relative prevalence reduction after 15 years using a proportional versus a most efficient injecting risk targeted treatment and vaccination strategy. *PWID* people who inject drugs

For a vaccine efficacy of 90 % and treatment or vaccination numbers of 10, 20 and 40/1,000 PWID per year, allocating treatments and vaccinations most efficiently in the model provided little additional benefit to 15-year relative prevalence reduction than proportional allocation (Fig. 3). These additional benefits were even smaller for lower vaccine efficacies.

### Reaching fixed prevalence reduction targets: simultaneously introducing a community vaccination programme to reduce treatment numbers

**Fig. 4:**
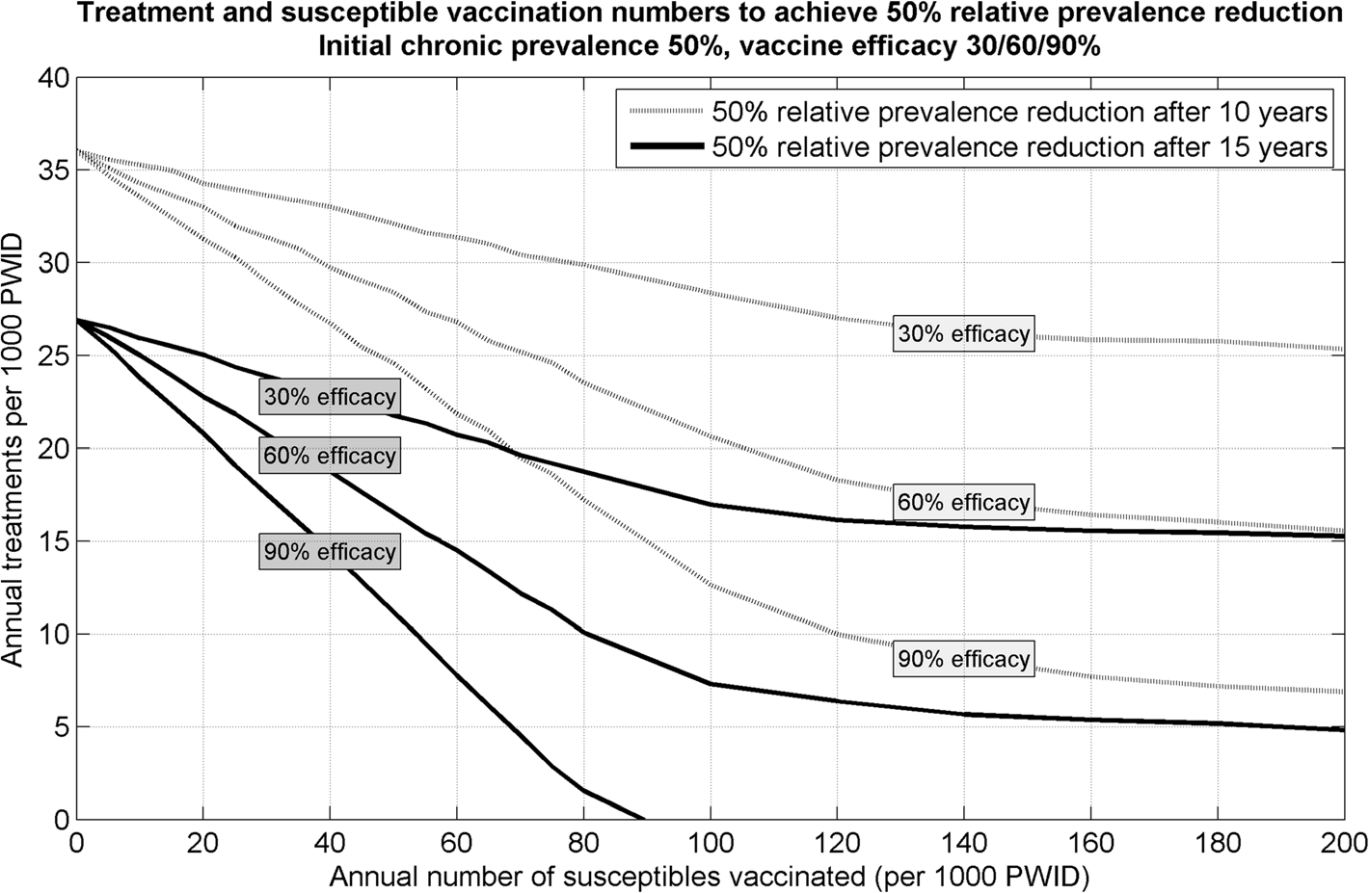
Achieving fixed targets: combinations of annual treatments and susceptible vaccinations that achieve a 50 % relative prevalence reduction target after 10 years (grey) and 15 years (black) with various vaccine efficacies (30, 60 and 90 %) for a 50 % initial chronic HCV prevalence among PWID. *HCV* hepatitis C virus, *PWID* people who inject drugs

The relationship between vaccination and treatment numbers that would achieve a 50 % relative prevalence reduction is approximately linear until allowed vaccination numbers become sufficient to cover the entire susceptible population (Fig. 4). For example, in a scenario with 50 % initial chronic prevalence, this is approximately 110 or 90/1,000 PWID each year for the 10 or 15 year models respectively. Allowing vaccination numbers beyond this only marginally alleviates the required treatment numbers—smaller additional benefits are the result of complete vaccination coverage being achieved sooner. Figure 4 also shows that in a setting with 50 % initial chronic HCV prevalence among PWID, a 90 % efficacious vaccine could achieve a 50 % relative prevalence reduction without any treatments by vaccinating approximately 90/1,000 PWID each year for 15 years.

For various scenarios, the annual numbers of susceptible vaccinations needed per unit of treatment withheld to maintain a specified reduction in prevalence (where vaccination numbers are insufficient to achieve complete coverage) are listed in Table 2. For example, in a setting with 50 % initial chronic HCV prevalence among PWID and a vaccine that was 60 % efficacious, instead of using treatment alone to achieve a 50 % relative prevalence reduction, substituting one annual treatment course for approximately five (4.8, see Table 2) annual susceptible vaccinations would result in the same relative prevalence reduction after 15 years, and greater reductions into the future.

**Table 2:** Achieving fixed targets: the annual number of susceptible PWID who must be vaccinated for every reduction in treatments administered in order to maintain a specified reduction in prevalence

		75 % relative prevalence reduction target			50 % relative prevalence reduction target			25 % relative prevalence reduction target		
	Time to target (years)	10	15	30	10	15	30	10	15	30
	*25 % initial chronic HCV prevalence among PWID*									
Vaccine efficacy	90 %	17.7	13.9	8.5	14.1	10.4	6.7	11.2	8.0	5.6
	60 %	26.8	21.3	14.1	21.2	15.5	10.0	17.0	12.1	8.5
	30 %	60.9	41.6	38.1	41.9	30.8	20.0	34.9	24.6	16.9
	*50 % initial chronic HCV prevalence among PWID*									
	90 %	5.8	4.3	2.9	4.3	3.2	2.1	3.4	2.4	1.6
	60 %	8.6	6.3	4.3	6.5	4.8	3.2	5.1	3.7	2.4
	30 %	17.2	13.0	8.9	13.0	9.7	6.5	10.3	7.5	4.9
	*75 % initial chronic HCV prevalence among PWID*									
	90 %	2.0	1.4	1.0	1.4	1.0	0.7	1.1	0.8	0.5
	60 %	3.0	2.1	1.5	2.2	1.5	1.0	1.6	1.1	0.8
	30 %	5.6	4.3	2.9	4.3	3.1	2.1	3.2	2.3	1.5

*HCV* hepatitis C virus, *PWID* people who inject drugs

Vaccinations become more effective at alleviating required treatment numbers when longer-term targets are used (see Additional file 1: Figure S2, left panel), and in settings where prevalence, and hence reinfection incidence [41], is high (see also Additional file 1: Figure S3). Vaccinations are slightly more effective at alleviating required treatment numbers for lower prevalence reduction targets, provided treatment numbers are insufficient to treat all infected PWID within the time frame (see Additional file 1: Figure S2, right panel).

For example, Melbourne, Australia is a setting with approximately 50 % chronic HCV prevalence among an estimated 25,000 PWID [42], and the proportion of PWID at high-risk, the average duration at high-risk, and length of injecting career parameters are suitable for this setting [25]. Figure 4 shows that to achieve a 50 % relative prevalence reduction after 15 years would require 26/1,000 PWID be treated each year, however if a 60 % efficacious vaccine were available, the same prevalence reduction target could be achieved with 22 treatments and 20 vaccinations per 1,000 PWID each year (i.e. Table 2 shows that approximately five vaccinations are required for every treatment reduction)—a reduction of approximately 100 treatment courses per year. Similarly, the model predicts that a 25 % relative prevalence reduction could be achieved by either treating 15/1,000 PWID each year, or treating 12/1,000 and vaccinating 12/1,000 PWID each year. After calibrating for an equivalent prevalence reduction with either treatment alone or treatment plus vaccination at 15 years, the treatment plus vaccination combination became more beneficial as time of follow-up increased. After 30 years, it leads to a substantially greater prevalence reduction.

### The basic reproduction number

For settings with 25, 50 and 75 % chronic HCV prevalence among PWID, the model estimated that the R0 values were 1.33, 2.08 and 4.33 respectively. In order to reduce R0 from above one to below one, it would be necessary to successfully vaccinate 24, 52 and 77 % of the susceptible population respectively, assuming PWID were vaccinated independently of risk (similar to the results in Fig. 3, individuals cycling through periods of high and low risk throughout their injecting career means that targeting vaccinations to specific risk groups has little effect on the required coverage). Achieving these levels of successful coverage would be difficult or impossible. For example, with a 60 % efficacious vaccine, the maximum successful coverage is 60 % (when all susceptible PWID are vaccinated), and so for the above example of Melbourne, coverage of 87 % would be required to reduce R0 to below one. Given the infrequent interaction of PWID with healthcare services, vaccinating high proportions of this population is likely to be difficult in practice. This indicates that further interventions, such as up-scaling NSPs, would be required to decrease the transmissibility of HCV so that R0 is less than one.

### Sensitivity of parameters

**Fig. 5:**
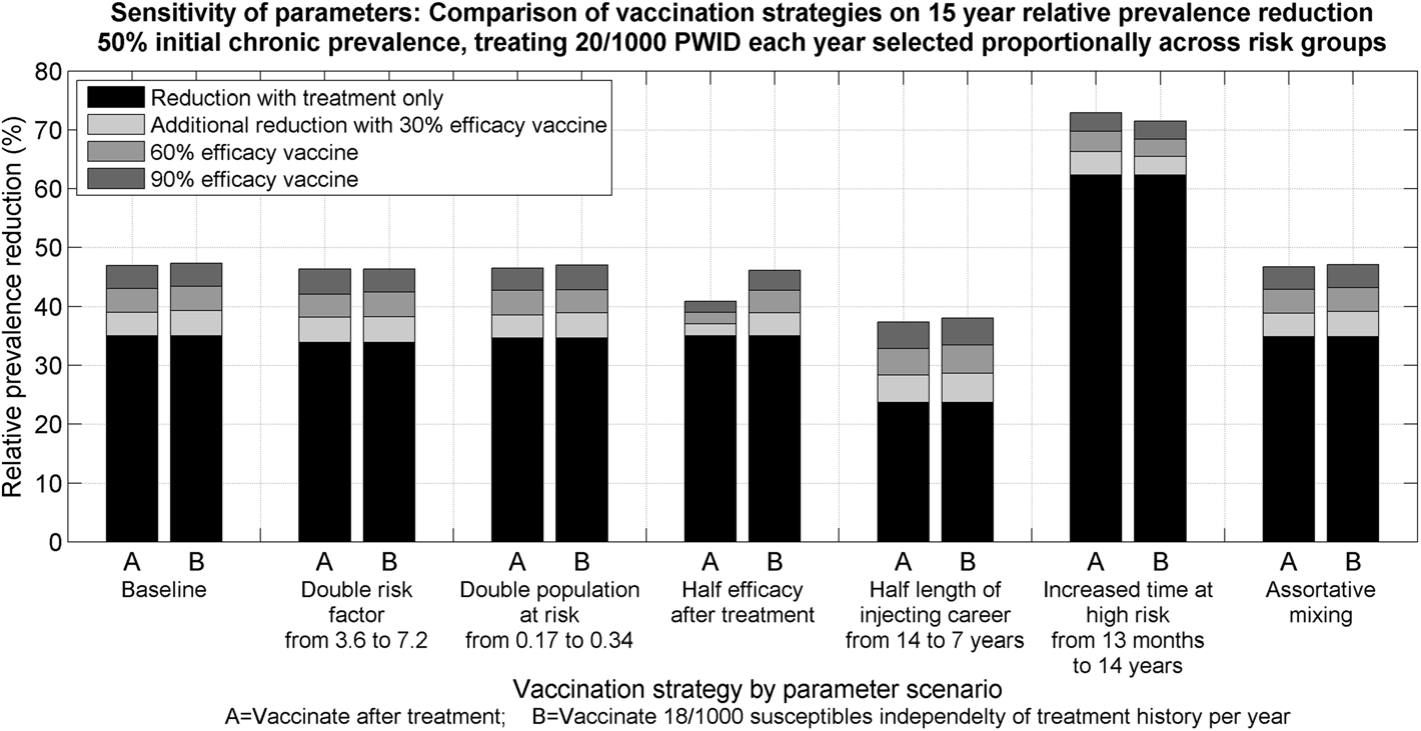
Sensitivity of vaccination strategy: comparison of 15-year relative prevalence reduction when vaccinating after treatment (*A*) versus vaccinating in the community under different parameter assumptions (*B*). The setting was with 50 % initial chronic HCV prevalence among PWID, treating 20/1,000 PWID each year, and using a 30, 60 or 90 % efficacious vaccine. *HCV* hepatitis C virus, *PWID* people who inject drugs

In a setting with 50 % chronic HCV prevalence among PWID, treating 20/1,000 PWID per year, and using a vaccine with 90 % efficacy, halving the vaccine efficacy after treatment (see ‘Methods’) led to vaccinating in the community having 12 % more total impact after 15 years than vaccinating after treatment (46 % versus 41 % relative prevalence reduction). Halving the length of injecting career from 14 years to 7 years led to vaccinating in the community having an additional 2.7 % impact after 15 years than vaccinating after treatment (38 % versus 37 % relative prevalence reduction). Increasing the duration spent at high risk from 13 months to 14 years led to vaccinating after treatment having 1.4 % more impact after 15 years than vaccinating in the community (73 % versus 72 % relative prevalence reduction). Otherwise, doubling the infectivity of the high-risk population from 3.6 to 7.2, doubling the proportion of PWID at high risk from 0.17 to 0.34, and assuming fully assortative mixing resulted in less than 1 % difference in 15-year relative prevalence reduction between vaccination strategies (Fig. 5).

**Fig. 6:**
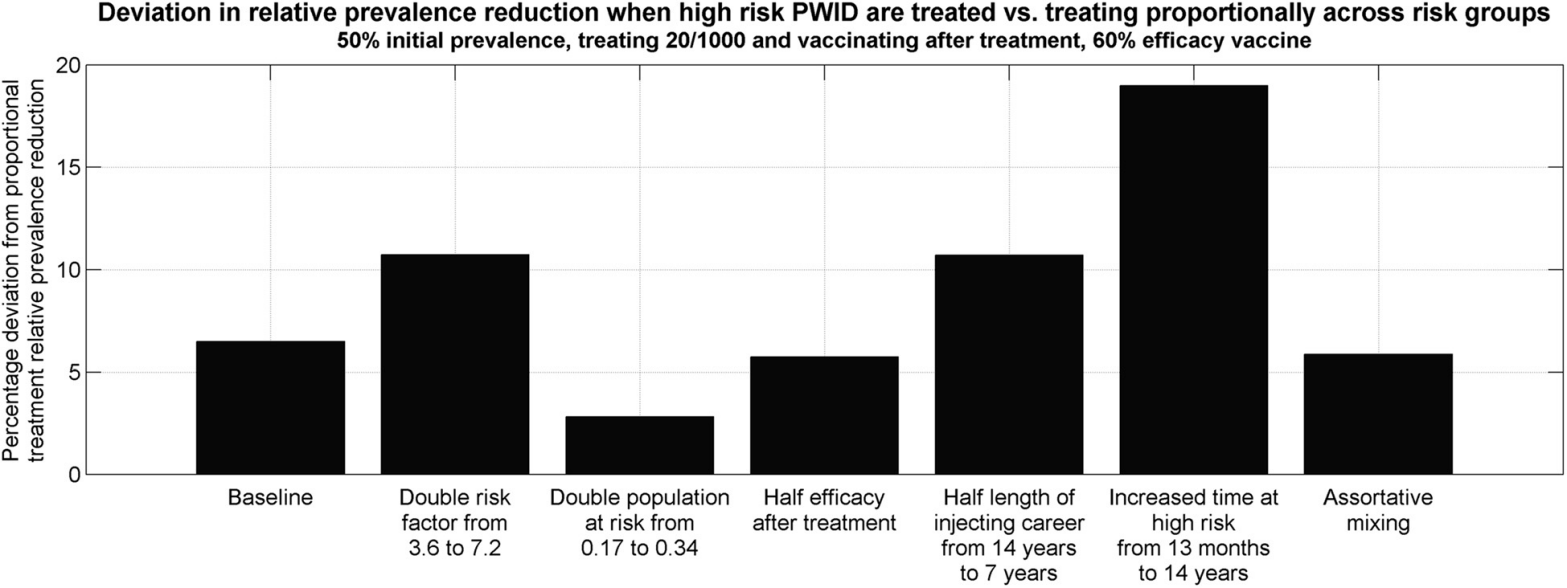
Sensitivity of risk allocation: the additional 15-year relative prevalence reduction that was possible by allocating treatments (20/1,000 PWID each year) most efficiently to risk groups and vaccinating after treatment in different parameter scenarios. The setting was with 50 % initial chronic HCV prevalence among PWID and a 60 % efficacious vaccine. *HCV* hepatitis C virus, *PWID* people who inject drugs

The additional benefit of treating risk groups efficiently was sensitive to the average duration at high risk. In a setting with 50 % chronic HCV prevalence among PWID, treating 20/1,000 PWID per year, and vaccinating after treatment with a 60 % efficacious vaccine, an improvement in 15-year prevalence reduction of 6 % could be achieved by allocating treatments to high-risk PWID rather than proportionally across risk groups; however, when the average duration at high risk was increased from 13 months to 14 years, this increased to 19 % additional impact (Fig. 6). As other parameters varied, the possible improvement in 15-year prevalence reduction changed to 11 % if the length of injecting career was halved from 14 to 7 years; 11 % if the infectivity of the high-risk group was doubled from 3.6 to 7.2; 6 % if vaccine efficacy was halved after treatment (see ‘Methods’); 6 % if fully assortative mixing was assumed; and 3 % when the high-risk population proportion was doubled from 0.17 to 0.34.

Quantitatively similar sensitivity results were found for different initial chronic prevalence, vaccine efficacy and treatment numbers, provided treatment numbers alone were insufficient to achieve complete coverage of the chronically infected population (approximately 20, 45 and 100/1,000 PWID per year for 15 years in settings with 25, 50 and 75 % initial chronic HCV prevalence among PWID respectively).

## Discussion

Previous models of HCV vaccines have either not considered concurrent vaccination and treatment [43–45] or have compared hypothetical vaccines to older generation therapies [46]. Here, we have explored the possible benefits of partially effective HCV vaccines alongside highly effective treatments. Our results suggest that even a partially effective HCV vaccine has benefits when used in conjunction with highly effective treatments in settings with medium (50 %) or high (75 %) chronic HCV prevalence among PWID. In these settings, prevalence reduction targets can be achieved with fewer treatment courses when a vaccination programme exists, which is likely to save on costs. For example, we estimated that in Melbourne, Australia, a 50 % relative prevalence reduction could be achieved with approximately 100 fewer treatment courses per year if a 60 % efficacious vaccine were administered after successful treatment. With treatment costs in developed countries (including Australia) still either in negotiation or unclear, and in some cases (e.g. the USA) up to US$80,000 a course, combining vaccination with treatment is likely to be a cheaper way to achieve prevalence reduction targets in settings with medium or high chronic HCV prevalence among PWID than treatment alone. In Melbourne, this could occur as long as treatment remains more than five times as expensive as a potential vaccine (Table 2). With vaccines for most other diseases costing under US$200 per person [47] this is not unrealistic, even if treatment costs are substantially reduced.

Administering vaccinations directly after successful treatment to prevent reinfection would be an efficient implementation strategy. Vaccinating directly after treatment achieved approximately equal additional prevalence reduction as vaccinating similar numbers in the PWID community, and, further, administering after treatment would be relatively simple because the person is already engaged in care with their HCV RNA status known. This would make simple implementation effective: in the previous example, treating 26/1,000 PWID per year would have equivalent effects to treating 22/1,000 PWID per year if vaccinating after treatment.

Background chronic prevalence was a more important determinant of a vaccine’s benefit than vaccine efficacy. In terms of reducing required treatment numbers, a vaccine with 30 % efficacy provided greater or equal benefits in a setting with high chronic HCV prevalence among PWID than a vaccine with 90 % efficacy in settings with low or medium chronic HCV prevalence among PWID. In many high-prevalence scenarios, a single vaccination provided almost the same benefit as a single treatment course. In these cases vaccinating after treatment would mean that treatment numbers could almost be halved with minimal effect on prevalence reduction.

The model had two main sensitivities. First, as the average length of injecting career increased so did the impact of treatments and vaccinations on prevalence reduction. This reflects the fact that in countries where longer injecting careers are typical, there is more time to accrue benefits through these interventions [9]. This is in contrast to harm reduction interventions (such as OST and NSPs), where modelling has shown reduced impact in settings with longer injecting careers owing to the long duration of coverage required to protect from infection [48]. Second, as the duration at high risk, and hence the heterogeneity of the population, increased, (1) the impact of treatments and vaccinations greatly increased, (2) vaccinating after treatment became an increasingly better strategy than community-based vaccination, and (3) the benefit of allocating treatments and vaccinations to specific injecting risk groups increased. The latter two changes were related: as the heterogeneity increased, the high-risk population in the model became more likely to be infected than the low-risk population and therefore more likely to be treated. This makes vaccination after treatment an implicitly more risk-targeted strategy, which in this case has increased benefits.

There is some concern that if people seeking treatment are not high-risk injectors, the benefits of vaccinating after treatment may be diluted. The model did not support evidence of this at baseline estimates for heterogeneity, because even the most efficient allocation of treatments and vaccinations across risk groups provided only small additional impact. However, because the benefits of risk-targeted treatment and vaccination strategies were sensitive to changes in duration at high risk, situation-specific data should be considered when forming HCV prevalence reduction strategies. For example, we found that in some settings, in the extreme case where the average time at high risk was the length of injecting career, up to 19 % additional relative prevalence reduction would be forgone if treatments were concentrated among low-risk compared to high-risk groups.

### Limitations and further work

These estimates are based on a theoretical model and there is uncertainty in the model parameters. Most importantly, we model a hypothetical vaccine of varying efficacy with perfect protection for the entire injecting career. This would require more than 14 years’ coverage, which is consistent with immunology researchers’ goals. Additionally, although most parameters come from observational studies, they are not specific to one location and may not be relevant to all PWID populations. However, we have explored a range of prevalence settings that we hope characterize the range of epidemics found. The model has assumed no differences between HCV genotypes when vaccinating or treating PWID, because immunology researchers are also aiming for cross-reactive immunity. Implementing combinations of treatment and vaccination numbers simultaneously without any scale-up period may be unrealistic; however, with a vaccine not yet developed and treatment currently available to the public, the approaches governments will take remain unclear.

This study only considers how the benefits of a vaccine vary by chronic HCV prevalence among PWID and under various treatment paradigms, and assumes that HCV is endemic before treatments and/or vaccinations are introduced. Further work should consider the history of HCV in particular locations and estimate these relationships using models that are calibrated to the corresponding epidemic curves. This would also allow estimation of how the transmission parameter π and R0 have changed over time as a result of changes to risk behaviour, and would improve estimation of the threshold number of susceptible PWID that would need to be vaccinated to reduce R0 to below one and lead to eventual elimination.

## Conclusion

Initial HCV prevalence has more impact on the overall effectiveness of a vaccine than vaccine efficacy. In a setting with high chronic HCV prevalence among PWID, even modest simultaneous coverage with a low-efficacy vaccine could significantly reduce the number of treatments required to achieve prevalence reduction targets. Administering vaccinations directly after successful treatment would be as effective as and more practical than vaccinating equal numbers of PWID in the community, and only minimal benefits could be gained by allocating treatments or vaccinations to specific injecting risk groups. Our results suggest that an HCV vaccine, even if only modestly efficacious, will play a key role in HCV elimination over the next 15–20 years.

## Additional file

Additional file 1:
**Additional charts (Figure S1.** Vaccination strategy by treatment number. **Figure S2.** Sensitivity of prevalence reduction target and time to measurement. **Figure S3.** Modelled chronic HCV prevalence among PWID), model equations and R0 calculations. (DOCX 866 kb)

## Abbreviations

DAAs: direct-acting antivirals
HCV: hepatitis C virus
NSPs: needle and syringe programmes
OST: opioid substitution therapy
PWID: people who inject drugs
SVR: sustained viral response
