# Supplementary material for: The role of a hepatitis C virus vaccine: modelling the benefits alongside direct-acting antiviral treatments
Source: BMC Med. 2015 Aug 20;13:198. doi: 10.1186/s12916-015-0440-2 (PMC4546023; doi:10.1186/s12916-015-0440-2)
Supplement: Additional file 1: — Additional charts (Figure S1. Vaccination strategy by treatment number. Figure S2. Sensitivity of prevalence reduction target and time to measurement. Figure S3. Modelled chronic HCV prevalence among PWID), model equations and R0 calculations. (DOCX 866 kb) [file 12916_2015_440_MOESM1_ESM.docx]

**Supplementary material**

The role of a hepatitis C virus vaccine: modelling the benefits alongside Direct-Acting Antiviral treatments

Nick Scott, Emma McBryde, Peter Vickerman, Natasha K Martin, Jack Stone, Heidi Drummer, Margaret Hellard.

**Additional charts**

Vaccinating after treatment was as effective at reducing prevalence as vaccinating an equivalent number of PWID in the community, and this remained true for other treatment numbers considered (Fig. S1).


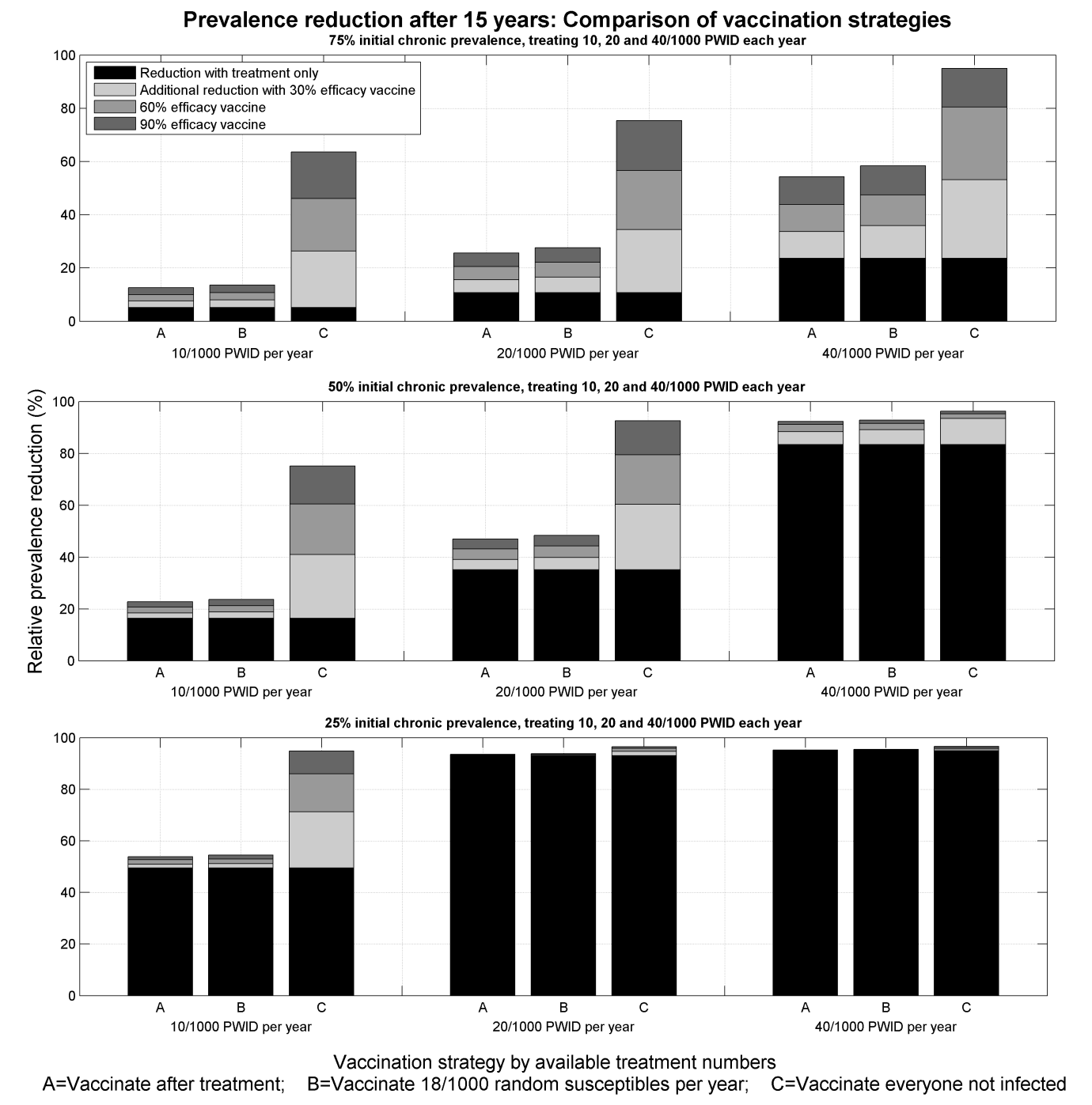


Fig. S1. Vaccination strategy by treatment number: Relative prevalence reduction with several vaccination strategies, for vaccine efficacies 90% (top), 60% (middle) and 30% (bottom).

Vaccinations would be most effective at alleviating required treatment numbers when longer term targets are used (Fig. S2, left panel), and would be slightly more effective at alleviating required treatment numbers when lower prevalence reduction targets are set (Fig. S2, right panel). In a setting with 50% initial prevalence, treatment numbers above approximately 45/1000 per year were sufficient to treat all chronically infected PWID within 15 years, and so increasing treatment numbers further cannot reduce prevalence (Fig. S2, right panel).


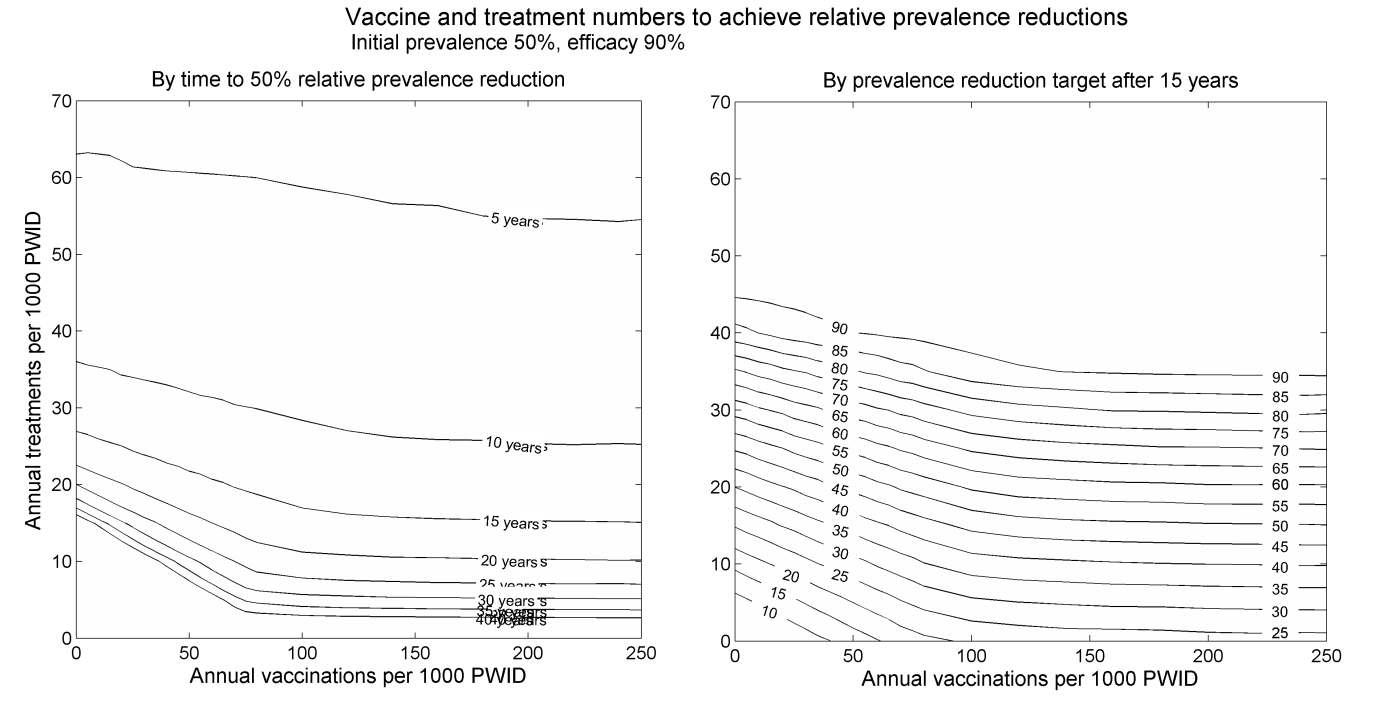


Fig. S2. Sensitivity of prevalence reduction target and time to measurement: How the relationship between treatment and vaccine numbers required to maintain prevalence reduction targets depends on how long before a target prevalence reduction is reached (left) and what the target prevalence reduction is (right).

Initial HCV prevalence had more impact on the overall effectiveness of a vaccine than vaccine efficacy; in particular, in a setting with high chronic HCV prevalence among PWID, even modest simultaneous coverage with a low-efficacy vaccine (i.e. from vaccinating after treatment) produced significant additional prevalence reduction (Fig. S3).


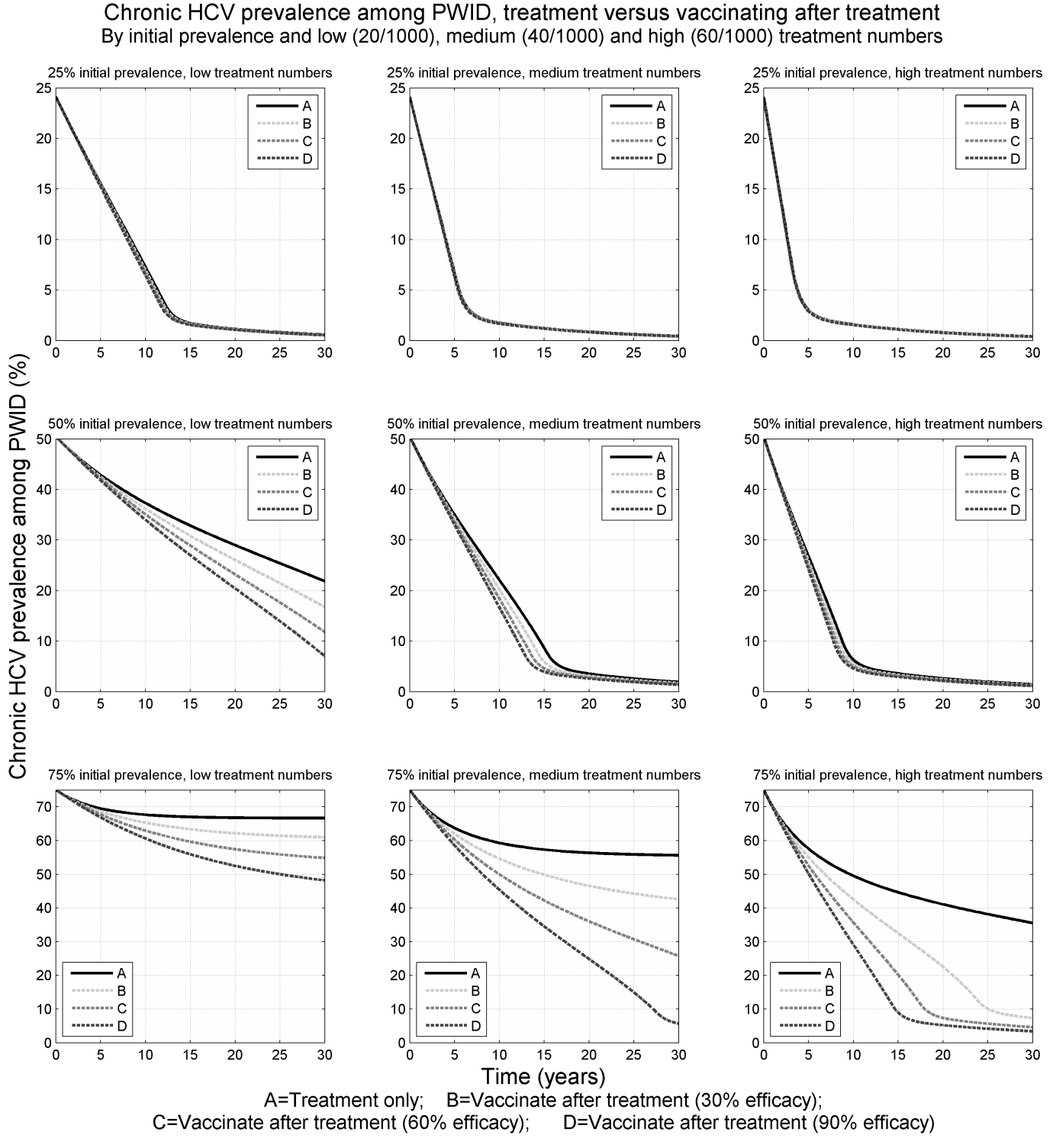


Fig. S3. Modelled chronic HCV prevalence among PWID: How prevalence changes over time with treatment only (solid line) compared to vaccinating after treatment (dashed lines; light, medium and dark shading indicating 30%, 60% and 90% efficacy vaccines respectively). Panels left to right indicate increasing treatment numbers (20, 40 or 60/1000 PWID per year respectively) and top to bottom indicates increasing initial chronic prevalence (25%, 50% and 75% respectively).

**Equations for high injecting risk group**

$$\frac{dS_{1}}{dt}=\mu N_{1}+\omega\alpha T_{1}+\eta S_{0}-\kappa S_{1}-\text{Ω}\left(V_{T},\omega\alpha T_{1}\right)-\text{Ω}\left(V_{S},S_{1}\right)-\pi\left(1-\delta\right)\text{ΨΓ}S_{1}-\mu S_{1}$$

$$\frac{dV_{1}}{dt}=\varepsilon\text{Ω}\left(V_{S},S_{1}\right)+\varepsilon\text{Ω}\left(V_{T},\omega\alpha T_{1}\right)+\eta V_{0}-\kappa V_{1}-\mu V_{1}$$

**Equations for low injecting risk group**

$$\frac{dS_{0}}{dt}=\mu N_{0}+\omega\alpha T_{0}-\eta S_{0}+\kappa S_{1}-\text{Ω}\left(V_{T},\omega\alpha T_{0}\right)-\text{Ω}\left(V_{S},S_{0}\right)-\pi\left(1-\delta\right)\text{Ψ}S_{0}-\mu S_{0}$$

$$\frac{dV_{0}}{dt}=\varepsilon\text{Ω}\left(V_{S},S_{0}\right)+\varepsilon\text{Ω}\left(V_{T},\omega\alpha T_{0}\right)-\eta V_{0}+\kappa V_{1}-\mu V_{0}$$

where Ψ and Ω are the risk weighted infectious population and treatment and vaccination number allocation respectively, defined below.

**Equation for the risk weighted infectious population**

**Equation for treatment and vaccination number allocation**

For an integer $x$ representing the number of treatments / vaccinations available and a compartment (or collection of compartments) of size$Y_{i}$:

$$\text{Ω}\left(x,Y_{i}\right)=\left\{\begin{matrix} x*\frac{Y_{i}}{Y_{0}+Y_{1}}, x\leq Y_{0}+Y_{1} \\ Y_{i}, \text{Otherwise} \end{matrix}\right.$$

**Total population in model**

*N=1000*

$$N_{1}=0.17*N$$

$$N_{0}=N-N_{1}$$

**Equations for the infectious population assuming fully assortative mixing**

Different representations for the infectious population are used for equations among the low and high risk injecting populations:

**Equations for the allocation of treatments and vaccinations to specific injecting risk groups**

If r is the proportion of treatments (or analogously vaccinations) to be allocated to high risk injectors, then different representations for the treatment and vaccination number allocation function Ω are used in the equations for high and low risk injectors:

$\text{Ω}\left(x,Y_{1}\right)=\left\{\begin{matrix} rx, rx<Y_{1} \text{and }\left(1-r\right)x<Y_{0} \\ rx+\left(\left(1-r\right)x-Y_{0}\right), rx<Y_{1} \text{and}\left(1-r\right)x\geq Y_{0} \text{and} x<Y_{1}+Y_{0} \\ Y_{1}, \text{wise} \end{matrix}\right.$*.*

$\text{Ω}\left(x,Y_{0}\right)=\left\{\begin{matrix} \left(1-r\right)x, rx<Y_{1} \text{and } \left(1-r\right)x<Y_{0} \\ \left(1-r\right)x+\left(rx-Y_{1}\right), \left(1-r\right)x<Y_{0} \text{and } rx\geq Y_{1} \text{and } x<Y_{1}+Y_{0} \\ Y_{0}, \text{Otherwise} \end{matrix}\right.$*.*

**The basic reproduction number**

We use the methods from Diekmann et al. (1)—see also (2)—to calculate the basic reproduction number (R0) of our system; the spectral radius (maximum of the absolute value of eigenvalues) of the next generation matrix. Let:

- $x=\left(S_{0}, S_{1},C_{0},C_{1},T_{0},T_{1},V_{0},V_{1},\hat{S}_{0},\hat{S}_{1},\hat{C}_{0},\hat{C}_{1},\hat{T}_{0},\hat{T}_{1},F_{0},F_{1}\right)^{T}$be the vector of compartments;
- be the rate of appearance of new infections in compartment k (i.e. individuals entering the kth element of x *only as a result of infection*);
- be the net rate individuals transfer out of compartment k by all means aside from new infection (i.e. is the number of individuals exiting compartment k minus the number of individuals entering compartment k, through means other than infection); and
- $x_{0}$ be the size of each compartment in the disease free equilibrium state.

At baseline (t=0), the only compartments with non-zero numbers of infectious individuals are C_0_ and C_1_ so that the relevant values are:

- ;
- ;
- ; and
- $x_{0}=\left(N_{0},N_{1},0,0,0,0,0,0,0,0,0,0,0,0,0,0\right)^{T}$ .

Then from (1, 2), R0 will be the spectral radius of

This was solved numerically in Matlab for settings with low (25%), medium (50%) or high (75%) chronic HCV prevalence among PWID—in each setting π is calibrated to a different value (0.0965, 0.1510 and 0.3141 respectively).

To determine the proportion of susceptibles that would need to be vaccinated to reduce R0 to below one—and hence have the most substantial effect on the epidemic—the size of each compartment in the disease free equilibrium state is altered to represent some proportion of the susceptible population, ζ, being vaccinated with a vaccine of efficacy ε. Thus $x_{0}=\left((1-\zeta)N_{0},(1-\zeta)N_{1},0,0,0,0,\zeta\varepsilon N_{0},\zeta\varepsilon N_{1},(1-\varepsilon)\zeta N_{0},(1-\varepsilon)\zeta N_{1},0,0,0,0,0,0\right)^{T}$and infections occurring in the components k=11 and k=12 of x must be considered; however, since the $(\hat{S},S)$ and $(\hat{C},C)$ compartments have identical infection dynamics, they can be added to simplify calculations. We then find ζ such that the spectral radius of

$$(1-\varepsilon\zeta)\frac{\pi(1-\delta)}{N_{0}+\text{Γ}N_{1}}\left[\begin{matrix} N_{0} & \text{Γ}N_{0} \\ \text{Γ}N_{1} & \text{Γ}^{2}N_{1} \end{matrix}\right]\left[\begin{matrix} \mu+\eta& -\kappa\\ -\eta& \mu+\kappa\end{matrix}\right]^{-1}$$

is less than one. This is equivalent to solving for , the proportion of the susceptible population who must be *successfully* vaccinated.

**References**

1. Diekmann O, Heesterbeek J, Metz JA. On the definition and the computation of the basic reproduction ratio R 0 in models for infectious diseases in heterogeneous populations. Journal of mathematical biology 1990;28:365-382.

2. Van den Driessche P, Watmough J. Reproduction numbers and sub-threshold endemic equilibria for compartmental models of disease transmission. Mathematical biosciences 2002;180:29-48.
